# Kinetic and structural insights into enzymatic mechanism of succinic semialdehyde dehydrogenase from *Cyanothece* sp. ATCC51142

**DOI:** 10.1371/journal.pone.0239372

**Published:** 2020-09-23

**Authors:** Congcong Xie, Zhi-Min Li, Fumei Bai, Ziwei Hu, Wei Zhang, Zhimin Li

**Affiliations:** 1 College of Bioscience and Bioengineering, Jiangxi Key Laboratory for Conservation and Utilization of Fungal Resources, Jiangxi Agricultural University, Nanchang, Jiangxi, China; 2 College of Science, Jiangxi Agricultural University, Nanchang, Jiangxi, China; Universidad de Santiago de Compostela, SPAIN

## Abstract

As a ubiquitous enzyme, succinic semialdehyde dehydrogenase contributes significantly in many pathways including the tricarboxylic acid cycle and other metabolic processes such as detoxifying the accumulated succinic semialdehyde and surviving in nutrient-limiting conditions. Here the *cce4228* gene encoding succinic semialdehyde dehydrogenase from *Cyanothece* sp. ATCC51142 was cloned and the homogenous recombinant cce4228 protein was obtained by Ni-NTA affinity chromatography. Biochemical characterization revealed that cce4228 protein displayed optimal activity at presence of metal ions in basic condition. Although the binding affinity of cce4228 protein with NAD^+^ was about 50-fold lower than that of cce4228 with NADP^+^, the catalytic efficiency of cce4228 protein towards succinic semialdehyde with saturated concentration of NADP^+^ is same as that with saturated concentration of NAD^+^ as its cofactors. Meanwhile, the catalytic activity of cce4228 was competitively inhibited by succinic semialdehyde substrate. Kinetic and structural analysis demonstrated that the conserved Cys262 and Glu228 residues were crucial for the catalytic activity of cce4228 protein and the Ser157 and Lys154 residues were determinants of cofactor preference.

## Introduction

Cyanobacteria are a kind of ancient prokaryotes which carry out oxygenic photosynthesis to change atmospheric chemistry and play an important role in aquatic ecosystems [1]. There are more than 2000 species in 150 genera of cyanobacteria with various shapes and sizes around the world [2]. *Cyanothece* sp. ATCC51142, one species of the genus *Cyanothece*, is the first genome sequenced diazotroph that can fix atmospheric nitrogen [3]. Cyanobacteria become attractive due to its capability of being genetically engineered to produce biofuels and other value-added products [4]. In addition, potent N_2_-fixing cyanobacteria are exploited as biofactory to produce feedstocks in the field of agriculture [5].

In canonical tricarboxylic acid (TCA) cycle, α-ketoglutarate (α-KG) is transformed into succinic acid through succinyl-CoA intermediate by two functional enzymes of α-KG dehydrogenase and succinyl-CoA synthetase. Unlike the canonical TCA cycle, α-KG is catalyzed by α-KG decarboxylase to generate succinic semialdehyde (SSA), which then is catalyzed by succinic semialdehyde dehydrogenase (SSADH) to produce succinic acid in many cyanobacteria because of the absence of key α-KG dehydrogenase [6, 7]. BLAST sequence analysis showed that the two genes of *cce4227* and *cce4228* encoded respective α-KG decarboxylase and SSADH in *Cyanothece* sp. ATCC51142. However, there is no detailed physiological and biochemical characterization of cce4227 and cce4228 proteins.

SSADH (EC 1.2.1.79) was an NAD(P)^+^-dependent oxidoreductase, one member of the aldehyde dehydrogenase superfamily. The oxidation of SSA was catalyzed by SSADH with NAD(P)^+^ as the cofactor to produce succinic acid, which is an intermediate in the TCA cycle. Therefore, SSADH played a vital role in metabolic pathways such as α-ketoglutarate shunt [8, 9], γ-aminobutyric (GABA) shunt [7, 10] and *p*-hydroxyphenylacetate shunt [11]. Once SSADH was missing in these TCA cycle variations, toxic substrates such as SSA, GABA and 4-hydroxybutyric acid (GHB) would accumulate in large quantities in cells, which could cause different degrees of harm in different organisms. In human, SSADH deficiency would lead to a rare hereditary neuropharmacological disorder, which was displayed by the clinical phenotype such as psychomotor arrest, developmental delay, language impairment, ataxia, lethargy and convulsion [12–14]. SSADH deficiency not only causes changes of development and phenotype in plants, but also makes plants sensitive to heat and UV radiation caused by the reactive oxygen intermediate [15, 16]. For bacteria, SSADH deficiency would lead to an imbalance of carbon and nitrogen metabolism in cells [17]. In addition, SSADH could promote the antioxidant defense ability of mitochondria by 4-hydroxynonenal which was the final product of oxidative peroxide lipid degradation [18].

Other than the physiological functional study of SSADH *in vivo*, there were many studies on its catalytical function and cofactor preference in recent years [19–22]. NADP^+^ or NAD^+^ was required as a hydride acceptor in the reaction of SSA to succinic acid catalyzed by SSADH. Initially, two genes of *YneI* and *GabD* were found in *Escherichia coli* to encode two types of SSADHs, which were dependent on NAD(P)^+^ and NADP^+^ cofactors, respectively [23]. As a result, SSADHs were categorized to two classes of YneI and GabD on account of cofactor preference. Generally, SSADHs with a small Ser residue in the active site preferred NADP^+^ cofactor since its active site could adapt the adenosine nucleoside phosphate group in NADP^+^ cofactor. SSADHs from *Streptococcus pyogenes* [24] and *Anabaena* sp. PCC7120 [25] were examples. On the other hand, SSADH from *Salmonella typhimurium* preferred NAD^+^ cofactor because its active center had a larger Lys160 residue [22], which could not accommodate the adenosine nucleoside phosphate group.

In this study, the detailed kinetic characterization and homology modeling of cce4228 protein from *Cyanothece* sp. ATCC51142 were carried out. Biochemical characterization revealed that cce4228 protein displayed optimal activity with MgCl_2_ at basic condition. Moreover, cce4228 protein showed same catalytic efficiency with saturated concentration of NADP^+^ or NAD^+^ as cofactor, although cce4228 protein had a higher binding affinity for NADP^+^ than NAD^+^. Site-directed mutagenesis demonstrated that the conserved Cys262 and Glu228 residues were critical for the enzymatic activity of cce4228 protein. Homology modeling revealed the structural basis of Ser157 and Lys154 residues to determine cofactor preference.

## Materials and methods

### Chemicals and reagents

*E*. *coli Trans*DH5α and BL21(DE3) competent cells, pET-28a vector were prepared and preserved by our lab. The genomic DNA of *Cyanothece* sp. ATCC51142 was purchased from American Type Culture Collection. DNA polymerase, DNA marker, *Nde*I and *Xho*I endonucleases, Fast Mutagenesis Kit were obtained from TransGen Biotech (Beijing, China). T4 DNA ligase, 2×Premix Taq and *Dpn*I were products of TaKaRa Biotechnology (Dalian, China).

### Overexpression and purification of cce4228 protein

Open reading frame of *cce4228* gene from *Cyanothece* sp. ATCC51142 (GenBank: ACB53576), encoding a succinic semialdehyde dehydrogenase, was amplified from the template of genomic DNA of *Cyanothece* sp. ATCC51142 with commercial primers (S1 Table in S1 File) by PCR. The constructed recombinant pET28a-*cce4228* plasmid was transformed into *Trans*DH5α competent cells and verified by DNA sequencing. Recombinant cce4228 protein with 6xHis at N-terminus was obtained as described previously [26]. Briefly, pET28a-*cce4228* plasmid transformed *E*. *coli* BL21(DE3) cells were grown at 25°C, 220 rpm in Luria broth (LB) media with 50 μg/mL kanamycin, then the cells were induced by 0.2 mM isopropyl-β-D-thiogalactopyranoside when *OD*_600nm_ of cells reached 0.8 and cultured for another 24 h at 25°C, 180 rpm. The cells were centrifuged and then lysed by sonication. Recombinant cce4228 protein were purified to be homogenous with Ni-NTA agarose resin and dialysis in 50 mM Tris-HCl, pH 8.0 was used to remove imidazole from the protein preparation. Mutants of cce4228 protein were obtained by performing the same protocol as the wild-type except that mutations in pET28a-*cce4228* plasmid were introduced by site-directed mutagenesis with commercial primers (S1 Table in S1 File) according to the reference [25].

### Catalytic activity determination of cce4228 protein

The catalytic activity of cce4228 protein was determined according to the procedures described in reference [25]. Briefly, specific amounts of NADP^+^, MgCl_2_ and purified cce4228 protein were incubated in 50 mM 2-(*N*-cyclohexylamino) ethanesulfonic acid (CHES), pH 9.0 buffer for 5 min at 25°C, and then various concentrations of SSA were added to the assay solution to start the reaction. Absorbance of reaction mixture at 340 nm was recorded to calculate reaction initial velocity. The obtained initial reaction velocities and concentrations of SSA substrate were fitted with the following equations by KaleidaGraph Software to obtain kinetic parameters. All measurements were repeated three times.

$$v=v_{\text{max}}\left[S\right]/\left(\left[S\right]+Km\right),\;\text{without}\;\text{inhibitor}$$ (1)

$$v=v_{\text{max}}/\left(1+K_{\text{m}}/\left[\text{S}\right]+[\text{S}]/K_{\text{i}}\right),\;\text{excess-substrate}\;\text{inhibition}$$ (2)

Where *v* was the calculated initial velocity, [S] was the concentration of substrate. *v*_max_, *K*_m_ and *K*_i_, indicating respective maximum reaction velocity, Michaelis-Menten constant and inhibitory constant of the substrate, were given by the software. The error bars indicated the standard errors of the mean (SEM) in this study.

### Effects of chemical reagents on the activity of cce4228 wild-type

The effects of MgCl_2_, ethylene diamine tetraacetic acid (EDTA), dithiothreitol (DTT) and β-mercaptoethanol (β-ME) on the catalytic activity of wild-type cce4228 protein were investigated by measuring the reaction initial velocities at presence of the above chemicals (2 mM) in reaction solution.

### Structure prediction and homology modeling for cce4228

The amino acid sequence of cce4228 protein and SSADHs from other sources were aligned using ClusltalW method in MEGA 6.0 software. The tertiary structure of cce4228 protein was simulated with the ternary complex of SSADH from *Synechococcus* sp. PCC7002 with NADP^+^ and SSA (PDB ID: 3VZ3) as template by Swiss-Model [27] and depicted by Pymol 1.503 software [28]. The SSADH protein in 3VZ3 is that of the non-catalytic Cys262Ala variant [19]. The online SAVES v5.0 server (https://servicesn.mbi.ucla.edu/SAVES/) was used to evaluate the quality of simulated three-dimensional structure of cce4228 protein.

## Results and discussion

### Purification and mass determination of recombinant SSADH

**Fig 1:**
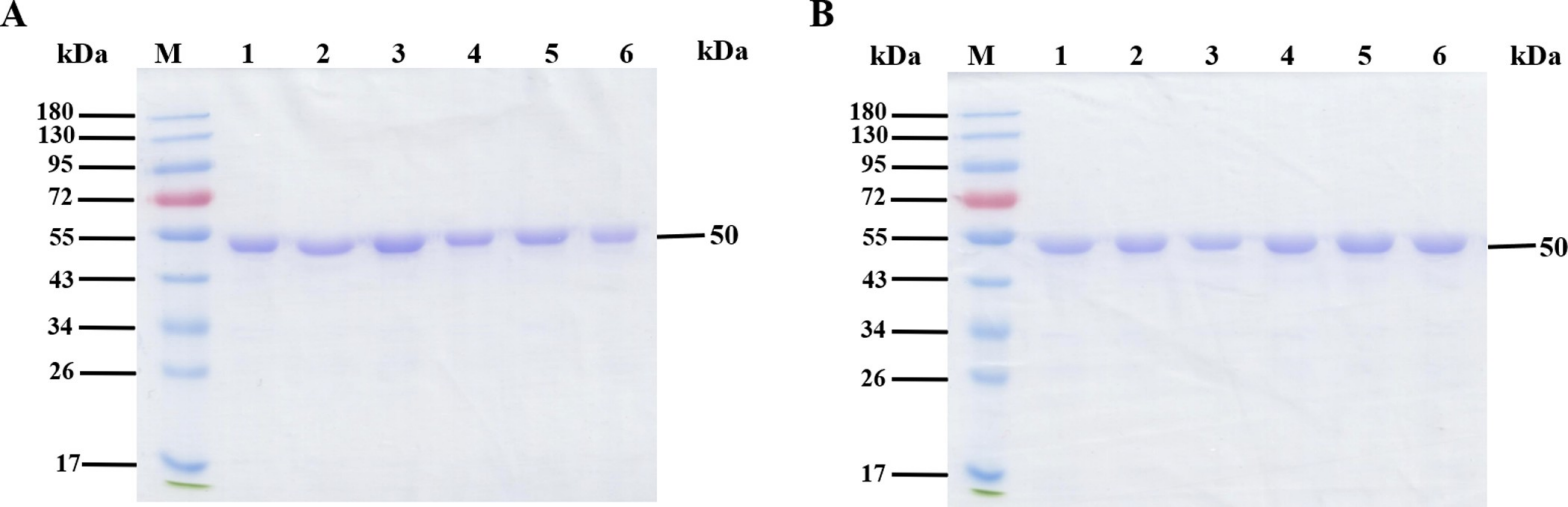
Purification of cce4228 wild-type and mutants. **A**. Lane M: protein marker; Lane 1–6: wild-type, Cys262Ala, Glu228Ala, Asn131Ala, Ser157Glu and Lys154Ala, respectively. **B**. Lane M: protein marker; Lane 1–6: Ser420Ala, Arg139Ala, Ser207Ala, Trp135Ala, Trp130Ala and Glu360Ala, respectively. 20 μg protein was deposited for both gels.

Open reading frame of *cce4228* gene encoding SSADH was cloned from the genomic DNA of *Cyanothece* sp. ATCC51142 successfully, and recombinant wild-type cce4228 protein was induced and overexpressed in *E*. *coli* BL21(DE3) [26]. After that, recombinant cce4228 protein was purified by fast protein liquid chromatograpy with Ni-NTA resin. The yield of homogenous cce4228 wild-type protein was 15 mg/g wet cells. SDS-PAGE estimated the approximate subunit molecular mass of cce4228 protein to be 50 kDa (Fig 1), which is close to the theoretical subunit mass of cce4228 protein with N-terminal 6xHis-tag (52518.13 Da). Meanwhile, the native mass of cce4228 protein was calculated to be *ca*. 109 kDa by size exclusion chromatography with standard protein ladders (S1 Fig in S1 File), which indicated that cce4228 protein was most probably a dimer. The oligomeric status of recombinant cce4228 protein was the same as that of SSADHs from *Synechococcus* sp. PCC7002 and *Anabaena* sp. PCC7120 [19, 20, 25]. The mutants of cce4228 protein were overexpressed and purified to be homogenous with close yield and purity as that of wide-type cce4228 (Fig 1).

### The effects of chemical reagents on catalytic activity of cce4228 wild-type

**Fig 2:**
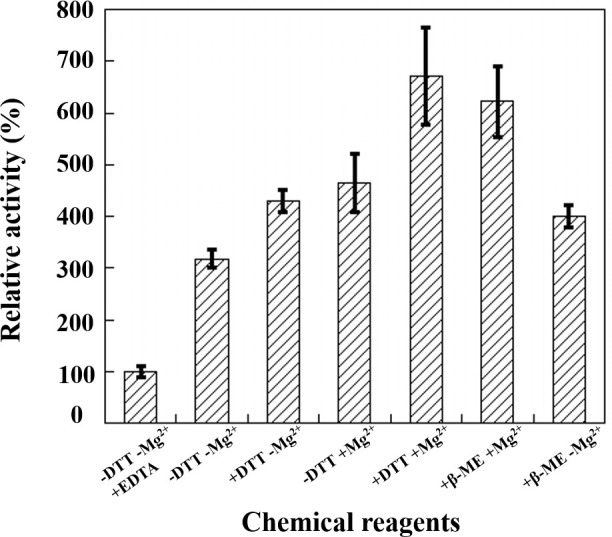
The effects of chemical reagents on catalytic activity of cce4228 wild-type protein. “+” indicates specific amounts of agents as indicated in the text were added to the assay solution, “-” indicates that agents not added to the assay solution. The catalytic initial velocity of cce4228 with 2 mM EDTA in assay solution was designated arbitrarily as 100% relative activity. The concentrations of cce4228 protein, SSA and NADP^+^ were fixed at 0.134 μM, 0.2 mM and 0.5 mM, respectively.

Previous results revealed that the catalytic activity of cce4228 protein would be either increased or decreased after adding metal ions into activity assay solution [26]. In this study, the activity of wild-type cce4228 protein would be decreased by 2-fold with the addition of 2 mM EDTA to remove the rudimental metal ions in assay buffer (the first bar *vs* the second bar) (Fig 2). This result indicated that the metal ion was important for cce4228 to display full function. As shown in Fig 2, the catalytic activity of cce4228 protein was elevated by 4~6 fold after adding 2 mM Mg^2+^ into assay buffer (the fourth, fifth or sixth bar *vs* the first bar). Meanwhile, the *K*_m_ value of Mg^2+^ and cce4228 protein was measured to be 6.24 μM [26], which could explain the low activity of cce4228 protein with 2 mM EDTA in the reaction solution. Since Cys residue in cce4228 protein was thought to function as a nucleophile in the catalytic mechanism, the reducing agent of DTT or β-ME was investigated whether it would affect the activity. The results showed that the reducing agents did not seem to have a considerable effect on the enzymatic activity of cce4228 protein in the optimal pH (the fifth or sixth bar *vs* the fourth bar in Fig 2). It indicated that the nucleophilic Cys residue was in the reduced state [29]. This indicative finding was confirmed by the fact that the Cys262 in the active site of cce4228 did not form a disulfide bond with other Cys residues, although there were six Cys residues in cce4228 protein (S2 Fig in S1 File).

### Cofactor preference of cce4228 protein

**Fig 3:**
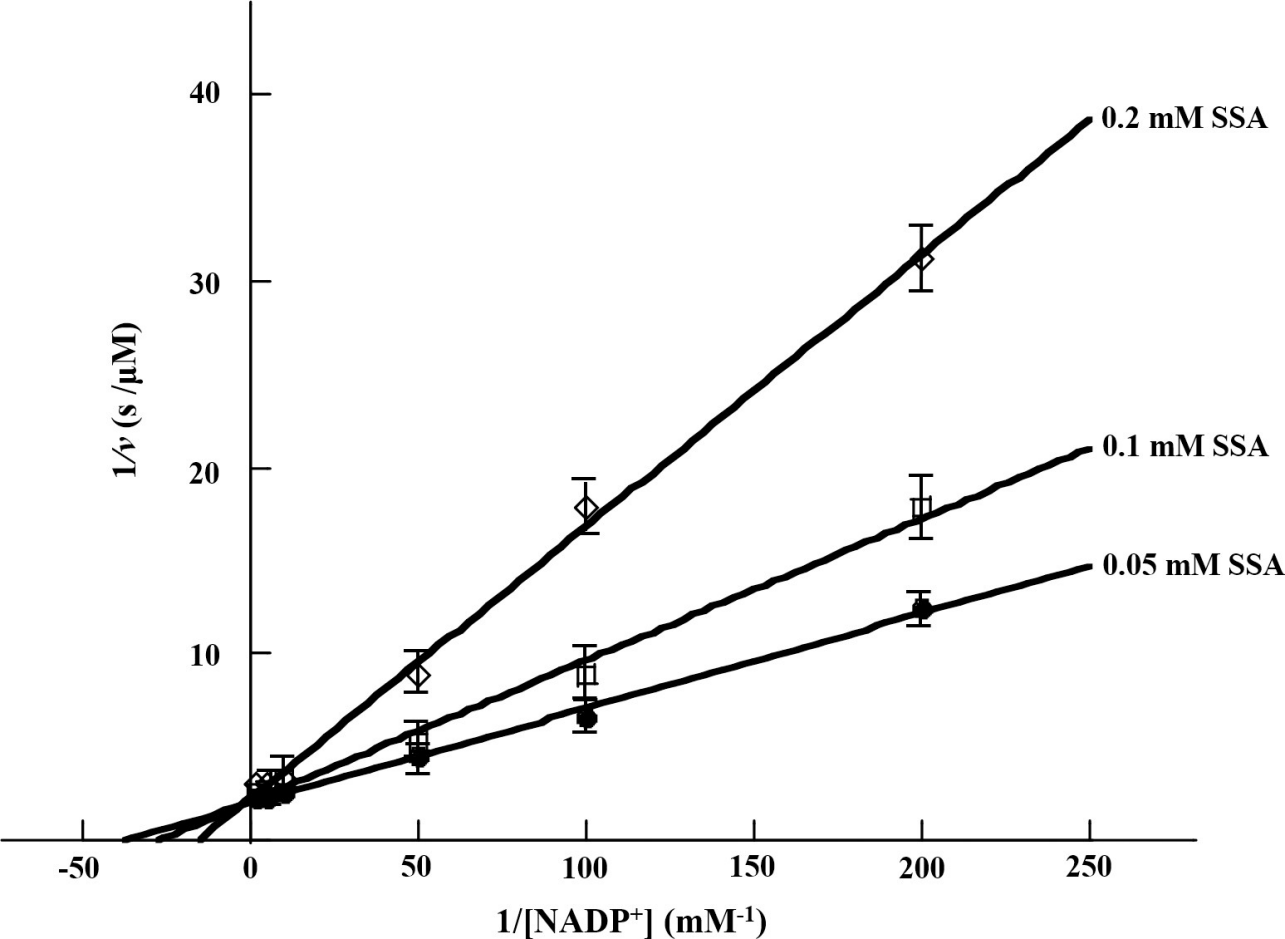
Lineweaver-Burk plot of cce4228 wild-type protein with NADP^+^ cofactor as substrate and SSA as inhibitor. The SSA concentrations were as labelled in the figure.

The amino acid sequence analysis revealed that cce4228 protein had Ser157 and Lys154 residues in its active center which might determine the cofactor preference (S3 Fig in S1 File). Meanwhile, the results of enzymatic kinetic experiments showed that cce4228 protein could use either NAD^+^ or NADP^+^ as cofactor (Table 1). The *K*_m_ values of cofactors with cce4228 were 1.7 mM and 0.034 mM for NAD^+^ and NADP^+^, respectively. Obviously, the binding affinity of NAD^+^ and cce4228 protein was *ca*. 50-fold lower than that of NADP^+^ and cce4228 protein if the *K*_m_ values were used as an estimation of the *K*_d_ values. However, cce4228 showed similar catalytic efficiency towards SSA at saturated concentration of NAD^+^ or NADP^+^. The *k*_cat_/*K*_m_ values of cce4228 wild-type were 487 mM^-1^s^-1^ and 435 mM^-1^s^-1^ with the cofactors of NADP^+^ and NAD^+^, respectively (Table 1). As a comparison, the catalytic efficiency of SSADH from *Anabaena* sp. PCC7120 towards SSA with NAD^+^ as cofactor was 8-fold lower than that with NADP^+^ as cofactor [25]. Furthermore, the kinetic pattern revealed that SSA substrate would inhibit the catalytic activity of cce4228 [26]. When the concentrations of SSA were increased to 0.02 mM and 0.1 mM with NAD^+^ and NADP^+^ as respective cofactors, the reaction rate of cce4228 would start to decrease [26]. The calculated inhibition constant (*K*_i_) values were 0.052 mM and 0.8 mM with NAD^+^ and NADP^+^ as cofactors, respectively (Table 1). Structural insight demonstrated that the SSA substrate inhibited the activity of SSADH from *Streptococcus pyogenes* by occupying the binding site of cofactor in the active center [30]. Actually, our kinetic results proved that SSA competitively inhibited the activity of cce4228 protein when NADP^+^ was used as cofactor (Fig 3). Since the binding affinity of NADP^+^ with cce4228 was 50-fold higher than that of NAD^+^ with cce4228, the inhibitive concentration of SSA to cce4228 wild-type was much higher with NADP^+^ as cofactor than that with NAD^+^ as cofactor. As a result, it looked like that NADP^+^ not NAD^+^ was the preferred cofactor for cce4228 wild-type protein.

**Table 1:** Kinetic parameters of cce4228 wild-type and mutants with different cofactors.

Cofactor	Parameters	wild-type^e^	Ser157Glu	Lys154Ala
NADP^+^	*K* m^SSA^ (mM) ^a^	0.008 ± 0.003	0.0028 ± 0.0007	0.02 ± 0.01
	*K* i ^SSA^ (mM) ^a^	0.8 ± 0.2	0.12 ± 0.02	0.02 ± 0.01
	*k*cat^SSA^ (s^-1^) ^a^	3.9 ± 0.3	1.5 ± 0.1	0.10 ± 0.05
	*k* cat/*K* m ^SSA^ (mM^-1^s^-1^) ^a^	487	535	5
	*K*NADP+ m (mM) ^b^	0.034 ± 0.002	1.1 ± 0.2	6.1 ± 1.5
NAD^+^	*K* m^SSA^ (mM) ^c^	0.004 ± 0.001	0.003 ± 0.001	0.02 ± 0.01
	*K* i ^SSA^ (mM) ^c^	0.052 ± 0.004	0.13 ± 0.03	0.02 ± 0.01
	*k* cat^SSA^ (s^-1^) ^c^	1.74 ± 0.08	0.58 ± 0.05	0.13± 0.07
	*k* cat/*K* m^SSA^ (mM^-1^s^-1^) ^c^	435	193	6.5
	*K*NAD+ m (mM) ^d^	1.7 ± 0.2	0.51 ± 0.04	1.5 ± 0.4

^a^ The concentrations of NADP^+^ were fixed at 0.5 mM, 10 mM and 8 mM for wild-type, Ser157Glu and Lys154Ala, respectively;

^b^ The concentrations of SSA were fixed at 0.2 mM, 0.02 Mm and 0.02 mM for wild-type, Ser157Glu and Lys154Ala, respectively;

^c^ The concentrations of NAD^+^ were fixed at 10 mM, 6 mM and 5 mM for wild-type, Ser157Glu and Lys154Ala, respectively;

^d^ The concentrations of SSA were fixed at 0.02 mM for all of wild-type, Ser157Glu and Lys154Ala;

^e^ data from ref. [26].

It was reported that the Ser157 of SSADH from *Synechococcus* sp. PCC7002 and *Anabaena* sp. PCC7120 functioned as cofactor preference determinant [20, 25]. To identify the residues involved in cofactor selectivity of cce4228 protein, the partial amino acid sequences of SSADHs from different sources were aligned (S3 Fig in S1 File). As shown in S3 Fig in S1 File, the corresponding residues of Ser157 in cce4228 were Glu231 and Glu225 in respective human SSADH and aldehyde dehydrogenase from *Arabidopsis thaliana*, both of which utilized NAD^+^ as their cofactor. Therefore, the cce4228 Ser157Glu mutant was constructed and the cofactor preference of Ser157Glu was further explored. The *K*_m_ value of NADP^+^ with Ser157Glu was 1.1 mM, whereas the *K*_m_ value of NAD^+^ with Ser157Glu was 0.51 mM (Table 1 and S4 Fig in S1 File). Compared to the binding affinity of wild-type cce4228 with cofactors, the binding affinity of Ser157Glu with NADP^+^ decreased by 30-fold (0.034 mM *vs* 1.1 mM), whereas the binding affinity of Ser157Glu with NAD^+^ increased by 3-fold (1.7 mM *vs* 0.51 mM) (Table 1). Obviously, the Ser157 residue of cce4228 protein functioned as a determinant of cofactor preference. Previous study showed that Lys160 residue in the active center determined the cofactor preference of SSADH from *Salmonella typhimurium*, which utilized NAD^+^ as its preferred cofactor [22]. The corresponding residue of Lys160 of SSADH from *Salmonella typhimurium* in cce4228 protein was Lys154 (S3 Fig in S1 File). Therefore, the Lys154Ala mutant was constructed and its cofactor preference was further investigated. Expectedly, Lys154Ala mutant displayed cofactor preference to NAD^+^ rather than NADP^+^. The *K*_m_ values of Lys154Ala with NAD^+^ and NADP^+^ were 1.5 mM and 6.1 mM, respectively (Table 1 and S5 Fig in S1 File). Interestingly, the binding affinity of cce4228 wild-type protein with NADP^+^ was much higher than that of Lys154Ala with NADP^+^ (0.034 mM *vs* 6.1 mM). It was worth pointing out that the *k*_cat_ values of Lys154Ala decreased dramatically with either NAD^+^ or NADP^+^ as cofactor, compared to the corresponding values of cce4228 wild-type, although the binding affinity of SSA with cce4228 wild-type or mutants did not change substantially. As a result, the catalytic efficiency of Lys154Ala was decreased by 100-fold to 5 mM^-1^s^-1^ with NADP^+^ as cofactor, compared to 487 mM^-1^s^-1^ for cce4228 wild-type (Table 1). It was the same case for Lys154Ala with NAD^+^ as cofactor (Table 1).

### The steady-state kinetic assay of cce4228 wild-type and mutants

**Fig 4:**
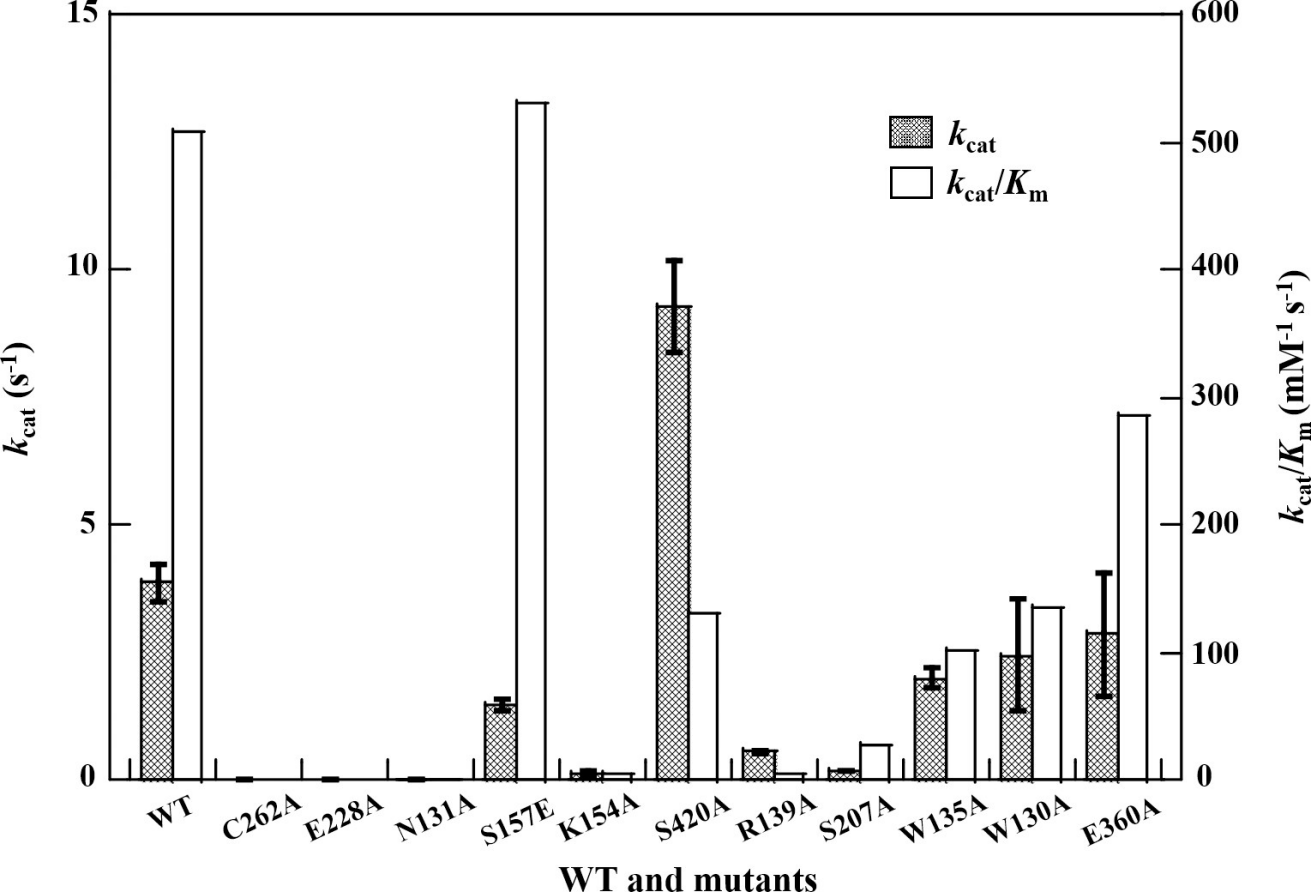
The *k*_cat_ and *k*_cat_/*K*_m_ values of cce4228 wild-type and mutants with NADP^+^ as cofactor.

To reveal the residues of cce4228 protein responsible for its catalytic activity, several residues in the enzyme active center or allosteric site were mutated. The Cys residue near the SSA substrate was believed to function as a nucleophile to attack SSA or NAD(P)^+^ as the first step in catalytic mechanism of SSADH [20, 30]. As a result, the cce4228 Cys262Ala mutant showed no activity to SSA (Fig 4 and S2 Table in S1 File). Meanwhile, Glu228Ala mutation rendered cce4228 inactive because the general base function by Glu228 vanished with this mutation. The Asn131Ala mutation would also inactivate cce4228 (Fig 4 and S2 Table in S1 File). Although the *k*_cat_ of Ser420Ala increased by 2-fold, the *K*_m_ of Ser420Ala with cce4228 increased by 9-fold. As a result, the catalytic efficiency (*k*_cat_/*K*_m_) of Ser420Ala was only 25% of that of cce4228 wild-type overall (Fig 4, S6 Fig and S2 Table in S1 File). Arg139Ala mutant showed substantially reduced binding affinity with SSA, whereas Ser207Ala had a similar binding affinity with SSA as that of cce4228 wild-type (S2 Table and S6 Fig in S1 File). These results indicated that Ser420 and Arg139 residues played important role in stabilizing SSA substrate. In addition, the mutations at Trp135, Trp130 and Glu360 would slightly affect their binding affinity to SSA or NADP^+^ (S2 Table and S6 Fig in S1 File). The catalytic efficiency of Trp135Ala, Trp130Ala and Glu360Ala reached 59%, 63% and 65% of that of cce4228 wild-type protein, respectively (Fig 4 and S2 Table in S1 File). Since all the aforementioned residues located in either SSA substrate binding pocket or NADP^+^ cofactor binding pocket, and the mutations at these residues would either affect the binding affinity or catalytic turnover rate, it was reasonable to conclude that there was synergistic effect between these residues in determining catalytic efficiency and cofactor preference.

### Homology modeling of wild-type cce4228 protein

**Fig 5:**
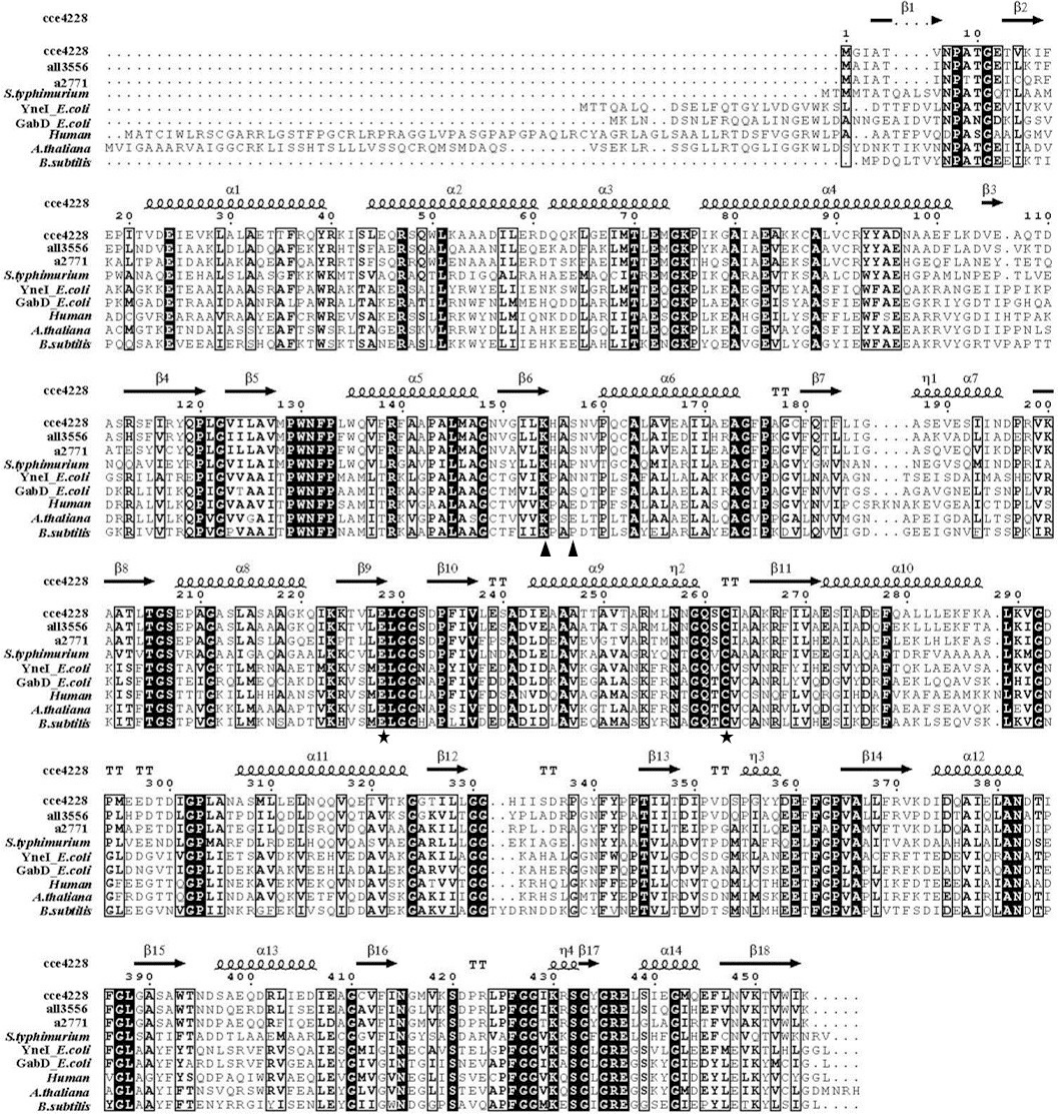
Amino acid sequence alignment of succinic semialdehyde dehydrogenases from different sources. SSADH sequences retrieved from the National Center for Biotechnology Information (NCBI): cce4228 protein from *Cyanothece* sp. ATCC51142 (ACB53576), all3556 from *Anabaena* sp. PCC7120 (BAB75255), a2771 from *Synechococcus* sp. PCC7002 (ACB00745), YneI from *Samlonella typhimurium* (NP_460484), YneI from *E*. *coli* (WP_115463367), GabD from *E*. *coli* (NP_417147), GabD from *Human* (NP_001071), GabD from *A*. *thaliana* (NP_178062), YneI from *Bacillus subtilis* (ARW30050). Triangle indicates residues involved in cofactor preference, and pentagram indicates residues involved in enzyme catalysis.

**Fig 6:**
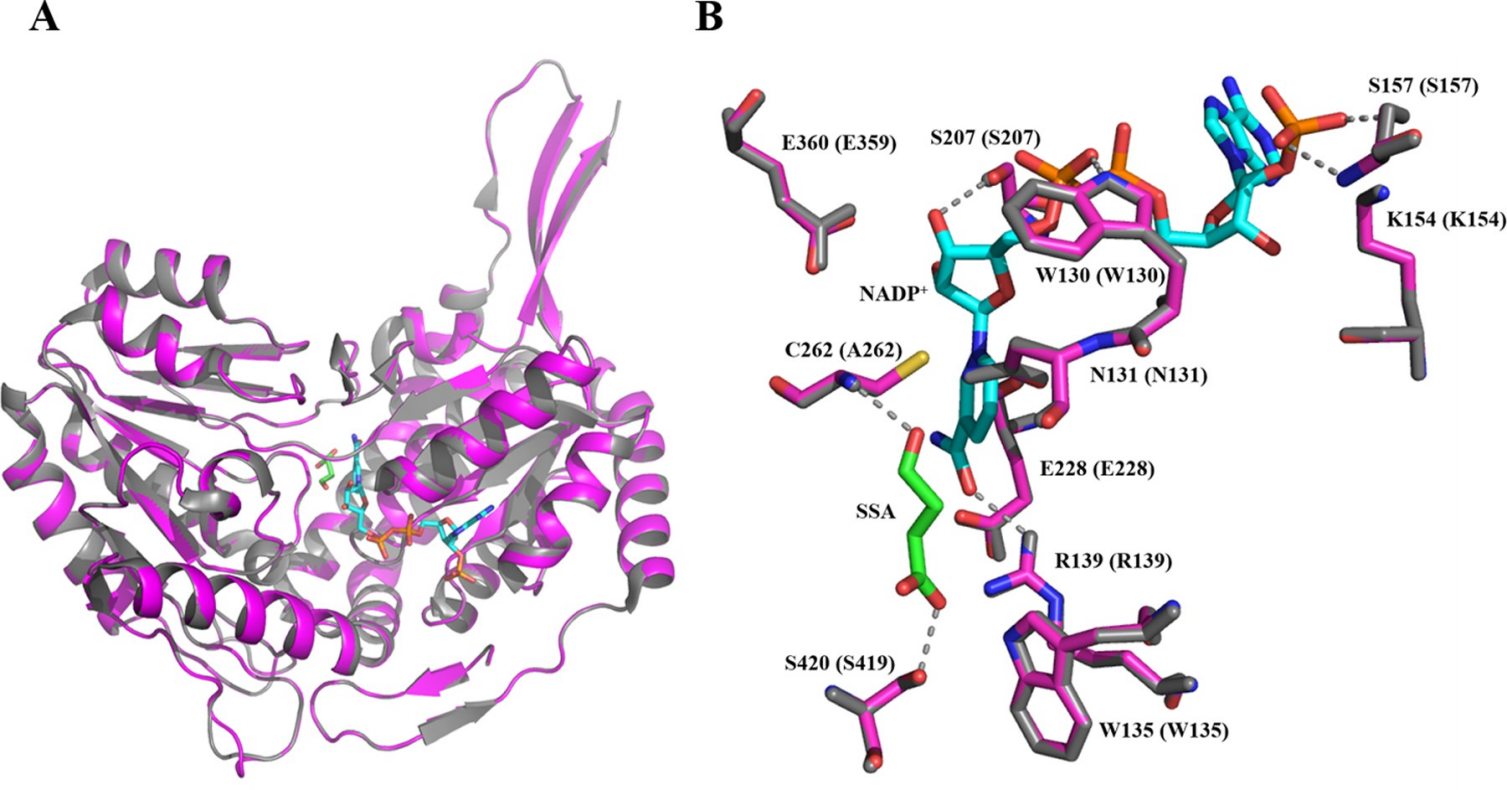
Structural modeling of cce4228 protein. **A.** The superposition of homology model of cce4228 protein and crystal structure of SSADH from *Synechococcus* sp. PCC7002 (PDB ID: 3VZ3), which were indicated by magenta and grey, respectively. The sticks were from the crystal structure with green (SSA) and cyan (NADP^+^) carbons. **B.** The close view of the active site. The residues with magenta and grey carbons were from homology model and crystal structure. Nitrogen, oxygen, phosphorus and sulfur atoms were colored as blue, red, orange and yellow, respectively. Residues were numbered according to the amino acid sequence of cce4228 protein. The numbers in parenthesis were from the amino acid sequence of crystal structure. The grey dashed lines indicated the possible interactions in the crystal structure. This figure was prepared by Pymol 1.503.

Based on phylogenetic analysis of cce4228 from *Cyanothece* sp. ATCC51142 with other SSADHs from different sources [26], we found that cce4228 protein was closely related to a2771 from *Synechococcus* sp. PCC 7002, whose crystal structure was available [19, 20]. The amino acid sequence identities between cce4228 and a2771 were 65% (Fig 5 and S7 Fig in S1 File). Therefore, the crystal structure of a2771 (PDB ID: 3VZ3) was used as template to construct the model structure of cce4228 by Swiss-Model and the model cartoon structure of cce4228 was displayed by Pymol 1.503 software. The model structure of cce4228 was almost overlapped with a2771 protein due to the high homology between cce4228 and a2271 (Fig 6). The Ramachandran plot showed that 93.6% of the residues of model structure of cce4228 were in the most favored regions and only 0.3% of the residues were in the disallowed regions, which indicated that the model structure of cce4228 was with great quality and high reliability (S8 Fig in S1 File). As shown in Fig 6B, the Ser157 and Lys154 residues of SSADH from *Synechococcus* sp. PCC 7002 were close to the adenosine nucleoside phosphate group of NADP^+^. The distances between the adenosine nucleoside phosphate group of NADP^+^ and the side chain OH group and the backbone NH group of Ser157 residue were only 2.6 Å and 2.9 Å, respectively. In addition, the distances between the side chain NH_3_^+^ group of Lys154 residue and the adenosine nucleoside phosphate group of NADP^+^ were in the range of 2.9–3.3 Å (Fig 6B). Once the Lys154 was mutated to Ala154 with short side chain, these interactions would be vanished. Since the Ser157 and Lys154 residues from homology model and crystal structure (PDB ID: 3VZ3) were almost overlapped, we speculated that the Ser157 and Lys154 residues from cce4228 protein would serve as determinants of cofactor preference. At the same time, this structural feature explained why the Ser157Glu and Lys154Ala mutations did not alter the binding affinity of cce4228 with SSA substrate. Meanwhile, the carboxyl group of SSA substrate was located in the vicinities of the side chains of Ser419, Trp135 and Arg139 in the crystal structure. As a comparison, the mutations of the corresponding residues in cce4228 protein of the above three residues resulted in dramatically reduced binding affinity of cee4228 protein with SSA (S2 Table in S1 File). Since Cys262 functioned as a nucleophile and Glu228 as a general base in catalytic mechanism, we could see that these two residues located near SSA and NADP^+^ (Fig 6B).

### Proposed enzymatic mechanism of cce4228 protein

**Fig 7:**
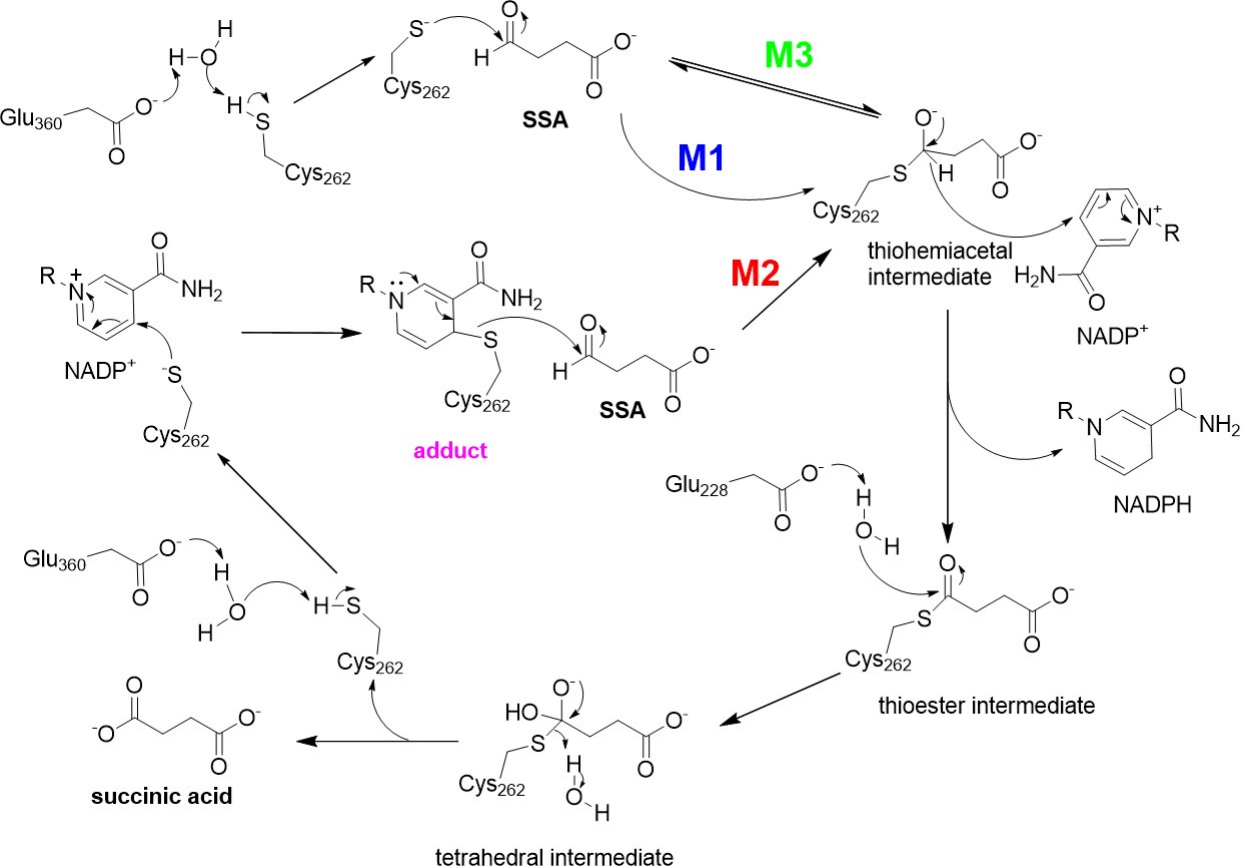
Proposed catalytic mechanisms for SSADHs from different sources. M1, M2 and M3 indicated the first, second and third mechanisms as stated in the text.

**Fig 8:**
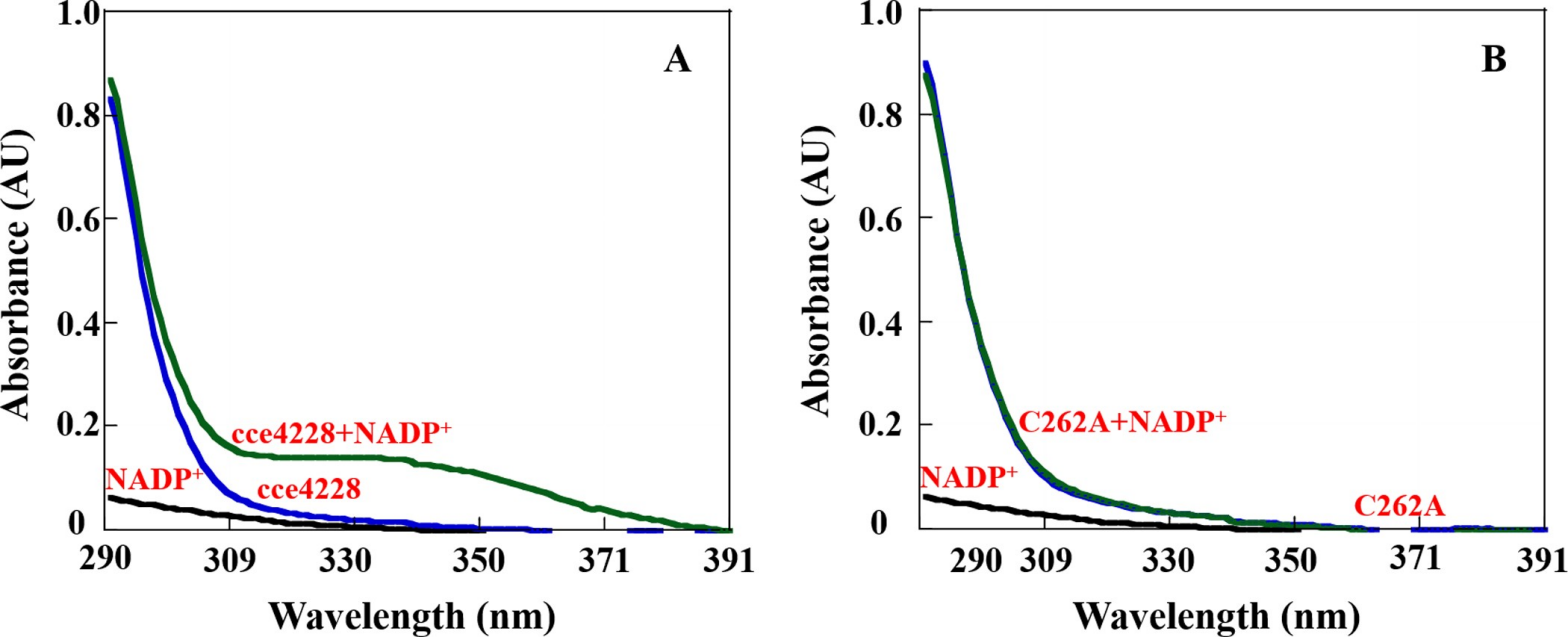
Spectrophotometric measurements of the cysteine-NADP^+^ adduct in solution using cce4228 wild-type and the Cys262Ala mutant. **A**. The absorbance of the cysteine-NADP^+^ adduct in solution using cce4228 wild-type. Spectra were recorded for 27 μM cce4228 (blue), 27 μM NADP^+^ (black), and a mixture of 27 μM cce4228 and NADP^+^ (green); **B**. The absorbance of the cysteine-NADP^+^ adduct in solution using the Cys262Ala mutant. Spectra were recorded for 27 μM Cys262Ala (blue), 27 μM NADP^+^ (black), and a mixture of 27 μM Cys262Ala and NADP^+^ (green).

The enzymatic mechanisms of SSADHs were well studied previously [19, 20, 22, 25, 30–32]. The key step of proposed catalytic mechanisms of SSADHs was to form the thiohemiacetal tetrahedral intermediate. However, there were several mechanisms for this formation of key intermediate (Fig 7). First, the nucleophilic Cys residue attacked the carbonyl group of SSA directly to form this key intermediate. SSADHs from *Streptococcus pyogenes* and *Salmonella typhimurium* were the examples [22, 30]. Second, the Cys residue in the catalytic center of SSADHs from cyanobacteria such as *Synechococcus* sp. PCC7002 [20] and *Anabaena* sp. PCC7120 [25] attacked NADP^+^ cofactor first to form cofactor-cysteine adduct, which might prevent the oxidation of catalytic cysteine. Then recruited SSA was attacked by the cofactor-cysteine adduct to form the key intermediate. Third, the SSADH from *Mycobacterium tuberculosis* adopted non-rapid equilibrium random mechanism to form thiohemiacetal tetrahedral intermediate [31]. In any case, the cysteine residue in the active site functioned as nucleophilic agent. SSADHs had two protection mechanisms to prevent the single catalytic Cys from being oxidized. Firstly, the key catalytic Cys forms disulfide bonds with non-catalytic Cys residues not far away from it to protect the key catalytic Cys from oxidation. It was the case for human SSADH [33]. Secondly, the key catalytic Cys by first attacking the cofactor NADP^+^ to form an E-NADP^+^ complex to prevent its key catalytic Cys from being oxidized [20, 25]. The cce4228 protein had only one Cys residue in the active center, therefore the redox-switch-mediated enzymatic mechanism was not appropriate for cce4228. To test whether cce4228 formed an adduct with NADP^+^ cofactor in reaction solution, spectrophotometric measurements were carried out. For the cce4228 wild-type, absorbance at the wavelength of 310~380 nm was observed clearly, which was generated by the proposed adduct [34] (Fig 8A). Since SSA substrate was not yet added into the assay solution, this absorption at 310~380 nm was not generated by NADPH, the reduced form of NADP^+^. Meanwhile, this absorption disappeared when Cys262Ala mutant substituted the cce4228 wild-type protein (Fig 8B). Thereby, cce4228 would adopt the second catalytic mechanism to generate the key thiohemiacetal tetrahedral intermediate. Briefly, Cys262 residue of cce4228 would first form an adduct with NADP^+^ and then this adduct would attack SSA substrate to form the key thiohemiacetal tetrahedral intermediate, which then was converted into thioester intermediate. With the help of Glu228 as a general base, the thioester intermediate would be hydrolyzed to succinic acid and release the protein. Basically, this mechanism was consistent with that of other SSADHs from cyanobacteria [19, 20, 25].

## Conclusions

The recombinant SSADH encoded by *cce4228* gene from *Cyanothece* sp. ATCC51142 was biochemically characterized in detail. Our results demonstrated that either NAD^+^ or NADP^+^ could be used as cofactor of cce4228 protein, although the binding affinity of NADP^+^ with cce4228 was much higher than that of NAD^+^ with cce4228. Meanwhile, SSA proved to be a competitive inhibitor of cce4228. Kinetic and structural analysis demonstrated that the conserved Cys262 and Glu228 residues were crucial for the catalytic activity of cce4228 protein and the Ser157 and Lys154 residues were determinants of cofactor preference. Finally, the enzymatic mechanism of cce4228 protein was suggested on account of kinetic data and model structure.

## Supporting information

S1 Raw images Raw gel images for Fig 1. (PDF)

S1 File (PDF)
