# Supplementary figures and images for: Kinetic and structural insights into enzymatic mechanism of succinic semialdehyde dehydrogenase from Cyanothece sp. ATCC51142
Source: PLoS One. 2020 Sep 23;15(9):e0239372. doi: 10.1371/journal.pone.0239372 (PMC7510979; doi:10.1371/journal.pone.0239372)

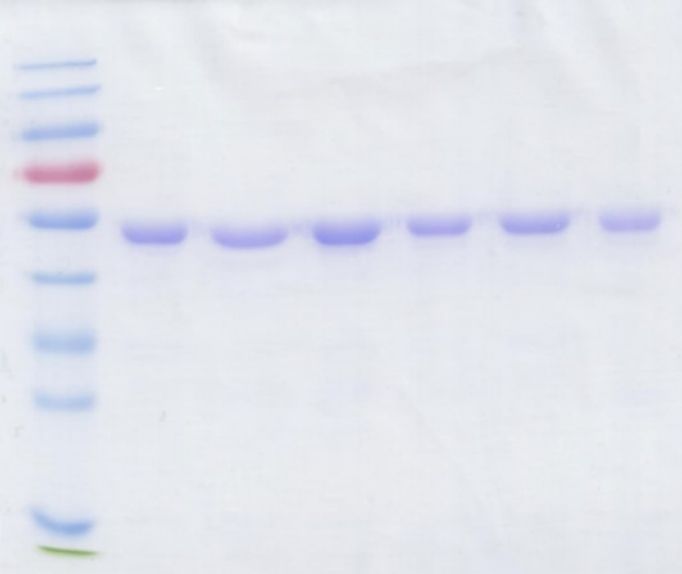

Supplement: S1 Raw images — (PDF) [file pone.0239372.s001.pdf]
