# Supplementary material for: Kinetic and structural insights into enzymatic mechanism of succinic semialdehyde dehydrogenase from Cyanothece sp. ATCC51142
Source: PLoS One. 2020 Sep 23;15(9):e0239372. doi: 10.1371/journal.pone.0239372 (PMC7510979; doi:10.1371/journal.pone.0239372)
Supplement: S1 File — (PDF) [file pone.0239372.s002.pdf]

# Kinetic and Structural Insights into Enzymatic Mechanism of Succinic Semialdehyde Dehydrogenase from *Cyanothece* sp. ATCC51142

Congcong Xie[1#], Zhi-Min Li[2#], Fumei Bai[1], Ziwei Hu[1], Wei Zhang[1], Zhimin Li[1*]

[1] College of Bioscience and Bioengineering, Jiangxi Key Laboratory for Conservation and Utilization of Fungal Resources, Jiangxi Agricultural University, Nanchang, Jiangxi, China

[2] College of Science, Jiangxi Agricultural University, Nanchang, Jiangxi, China

* Corresponding author

E-mail: zhiminli@jxau.edu.cn (ZL)

[#]These authors contributed equally to this work.

**S1 Table.** Primers sequences used to construct wild type and variant plasmids

| Variants | Primers Name | Primers Sequences |
|---|---|---|
| WT | cce4228-F | TGTAACT***CATATG***GGTATCGCTACAGTTAACCC |
| | cce4228-R | TAT***CTCGAG***TCACTTGATCCATACGGTTTTTAC |
| C262A | C262A-F | GAACAATGGTCAGTCTGCGATTGCTGCGAAAC |
| | C262A-R | CGCAGACTGACCATTGTTCAACATCCTAGCAG |
| E228A | E228A-F | AAGAAAACCGTTTTAGCGTTAGGGGGTAGC |
| | E228A-R | CGCTAAAACGGTTTTCTTAATCTGTTTACCGGC |
| N131A | N131A-F | GCCGTGATGCCCTGGGCGTTTCCCCTGTG |
| | N131A-R | CGCCCAGGGCATCACGGCTAAAATGACCCCT |
| S157E | S157E-F | GTATTCTCAAACACGCCGAAAACGTCCCTCA |
| | S157E-R | TTCGGCGTGTTTGAGAATACCCACATTACCC |
| K154A | K154A-F | GTAATGTGGGTATTCTCGCGCACGCCTCTAAC |
| | K154A-R | CGCGAGAATACCCACATTACCCGCCATTAATGC |
| R139A | R139A-F | CTGTGGCAAGTGTTTGCCTTTGCTGCC |
| | R139A-R | GCAAACACTTGCCACAGGGGAAAATTCC |
| S420A | S420A-F | TTATCAATGGGATGGTCAAAGCTGACCCCCG |
| | S420A-R | CTTTGACCATCCCATTGATAAAGACACATCCAGC |
| W135A | W135A-F | CCTGGAATTTTCCCCTGGCGCAAGTGTTTCG |
| | W135A-R | GCCAGGGGAAAATTCCAGGGCATCACGG |
| M128A | M128A-F | GGTCATTTTAGCCGTGGCGCCCTGGAATTTTC |
| | M128A-R | GCCACGGCTAAAATGACCCCTAAAGGTTGATAACG |
| S207A | S207A-F | CTGCTACTTTAACAGGAGCCGAACCCGCAG |
| | S207A-R | GCTCCTGTTAAAGTAGCAGCCTTAACTCTGGGATC |
| W130A | W130A-F | CATTTTAGCCGTGATGCCCGCGAATTTTCCCCTG |
| | W130A-R | GCGGGCATCACGGCTAAAATGACCCCTAAAGG |
| E360A | E360A-F | CACCTGGTTATTACGACGCGTTCTTTGGTC |
| | E360A-R | CGCGTCGTAATAACCAGGTGAATCTACAGG |

**S2 Table.** Kinetic parameters of cce4228 wild-type and mutant proteins.

| Proteins | Location of residues | $K_m^{SSA}$ (mM)[a] | $K_i^{SSA}$ (mM)[a] | $k_{cat}^{SSA}$ ($s^{-1}$)[a] | $k_{cat}^{SSA}$/ $K_m^{SSA}$ ($mM^{-1}s^{-1}$)[a] | $K_m^{NADP+}$ ( mM)[b] |
|---|---|---|---|---|---|---|
| wild-type[c] | / | 0.008 ± 0.003 | 0.8 ± 0.2 | 3.9 ± 0.3 | 487 | 0.034±0.002 |
| C262A | SSA | NA | NA | NA | NA | NA |
| E228A | $NADP^+$ | NA | NA | NA | NA | NA |
| N131A | $NADP^+$ | NA | NA | NA | NA | NA |
| S420A | SSA | 0.07 ± 0.01 | 0.7 ± 0.1 | 9.3 ± 0.9 | 133 | 0.025 ± 0.002 |
| R139A | SSA/$NADP^+$ | 0.112 ± 0.008 | 10.7 ± 1.5 | 0.54 ± 0.01 | 4.8 | 0.023 ± 0.004 |
| S207A | $NADP^+$ | 0.006 ± 0.003 | 0.6 ± 0.3 | 0.17 ± 0.02 | 28.3 | 0.008 ± 0.002 |
| W135A | SSA | 0.0195 ± 0.006 | 0.6 ± 0.1 | 2.0 ± 0.2 | 102 | 0.018 ± 0.001 |
| W130A | $NADP^+$ | 0.02 ± 0.01 | 0.05 ± 0.03 | 2.4 ± 1.0 | 120 | 0.024 ± 0.001 |
| E360A | $NADP^+$ | 0.010 ± 0.007 | 0.05 ± 0.03 | 2.8 ± 1.2 | 280 | 0.139 ± 0.009 |

NA: No Activity.

a. Varied concentrations of SSA with $NADP^+$ concentration fixed at 0.5 mM;

b. Varied concentrations of $NADP^+$ with SSA concentrations fixed at 0.2 mM for wild-type, S420A and W135A, 1 mM for R139A, 0.05 mM for S207A, 0.02 mM for W130A and E360A, respectively;

c. data from ref. 26.

## Supplementary Figures

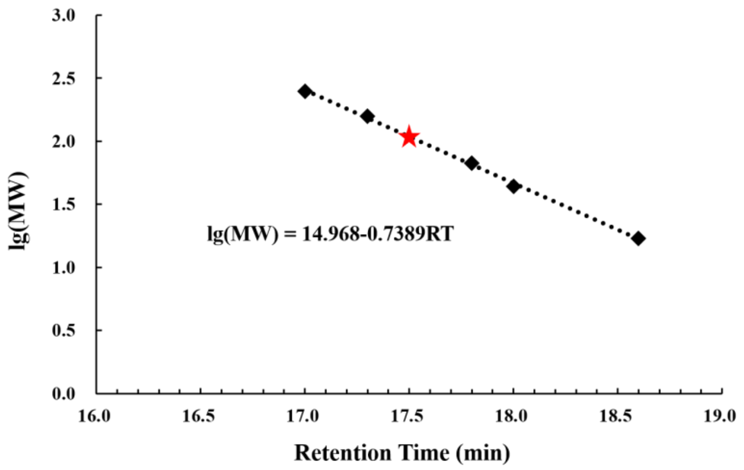


**S1 Fig.** The native mass determination of cce4228 wild-type protein. The used protein ladders were 17 kDa, 44 kDa, 67 kDa, 158 kDa and 250 kDa. The retention time of cce4228 was 17.5 min. Therefore, the native mass of cce4228 was calculated to be 109 kDa according to the formula.

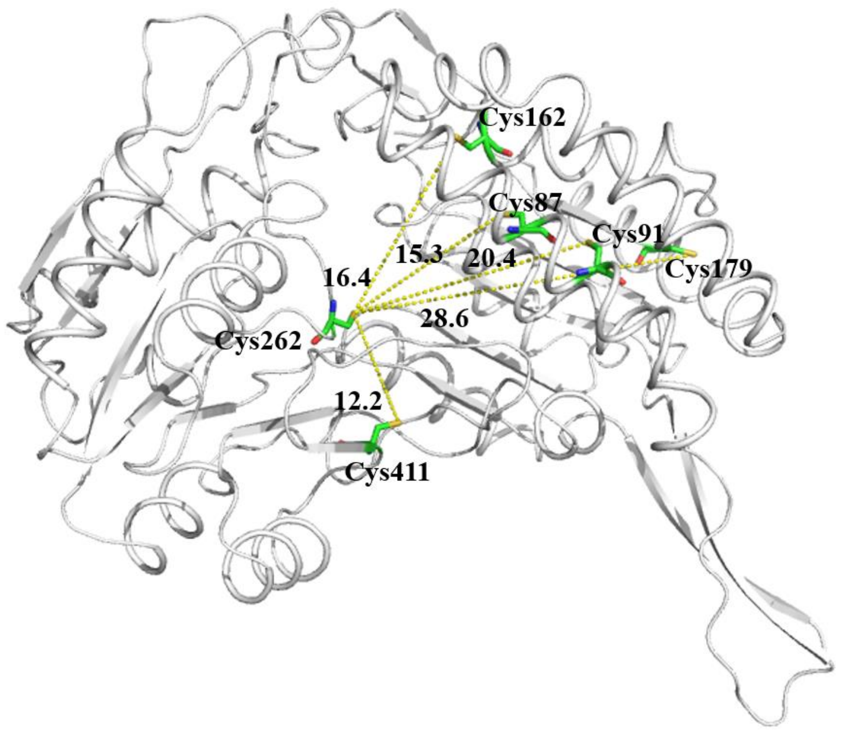


**S2 Fig.** The model cartoon structure of cce4228 protein. As shown in model structure, the Cys262 residue at the catalytic site was far from other Cys residues in cce4228 protein. The labelled value in angstrom indicated the distances between two sulfur atoms displayed as dashed yellow lines.

| | Key position of cofactor recognition | | | Cofactor preference |
|---|---|---|---|---|
| **cce4228** | 141 | AAPALMAGNVGLLKHASNVPQC | 162 | $NADP^+$ |
| **all3556** | 141 | AAPALMAGNVGLLKHASNVPQC | 162 | $NADP^+$ |
| **a2771** | 141 | AAPALMAGNVAVLKHASNVPQC | 162 | $NADP^+$ |
| **GabD_*E.coli*** | 167 | AGPALAAGCTMVLKPASQTPFS | 188 | $NADP^+$ |
| ***Human*** | 215 | VGAALAAGCTVVVKPAEDTPFS | 236 | $NAD^+$ |
| ***A.thaliana*** | 209 | VGPALASGCTVVVKPSELTPLT | 230 | $NAD^+$ |

**S3 Fig.** Alignment of amino acids involved in cofactor recognition of cce4228 protein and SSADHs from various organisms. The above organisms contained: cce4228 protein from *Cyanothece* sp. ATCC51142 (ACB53576), all3556 from *Anabaena* sp. PCC7120 (BAB75255), a2771 from *Synechococcus* sp. PCC7002 (ACB00745), GabD from *E. coli* (NP_417147), SSADH from *Human* (NP_001071), SSADH from *A. thaliana* (NP_178062).

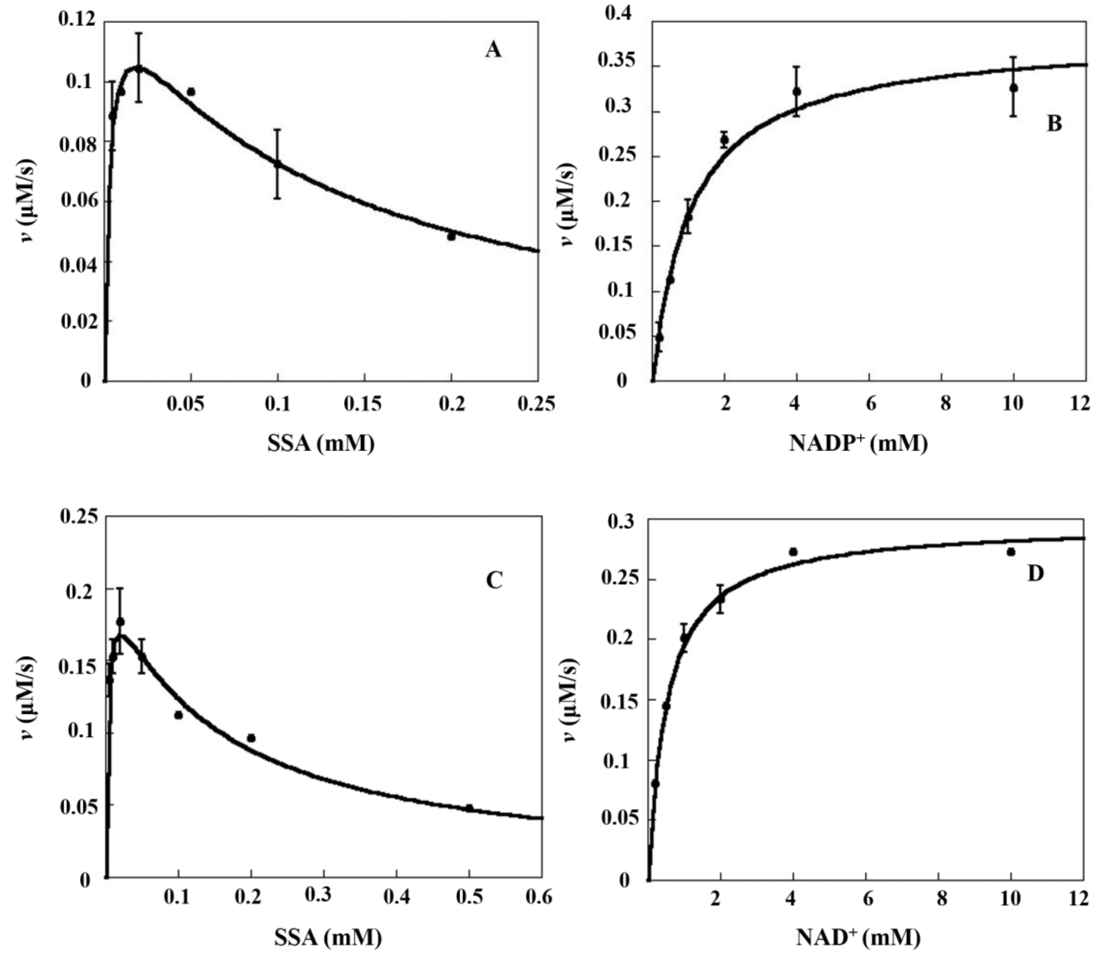


**S4 Fig.** The kinetic analysis of S157E mutant.

A: The initial velocities as function of SSA concentrations with the concentration of $NADP^+$ fixed at 10 mM;

B: The initial velocities as function of $NADP^+$ concentrations with the concentration of SSA fixed at 0.02 mM;

C: The initial velocities as function of SSA concentrations with the concentration of $NAD^+$ fixed at 6 mM;

D: The initial velocities as function of $NAD^+$ concentrations with the concentration of SSA fixed at 0.02 mM;

The above experiments were repeated 3 times.

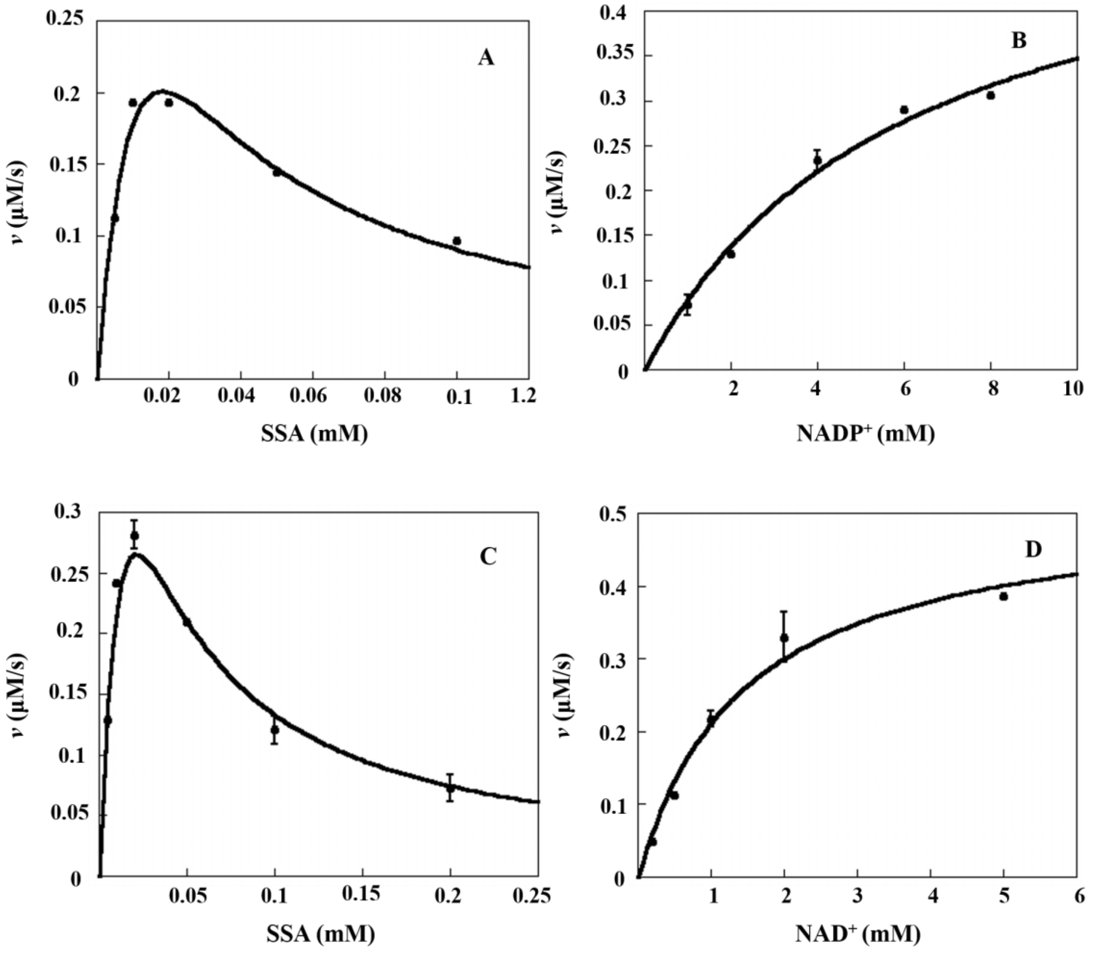


**S5 Fig.** The kinetic analysis of K154A mutant.

A: The initial velocities as function of SSA concentrations with the concentration of $NADP^+$ fixed at 8 mM;

B: The initial velocities as function of $NADP^+$ concentrations with the concentration of SSA fixed at 0.02 mM;

C: The initial velocities as function of SSA concentrations with the concentration of $NAD^+$ fixed at 5 mM;

D: The initial velocities as function of $NAD^+$ concentrations with the concentration of SSA fixed at 0.02 mM;

The above experiments were repeated 3 times.

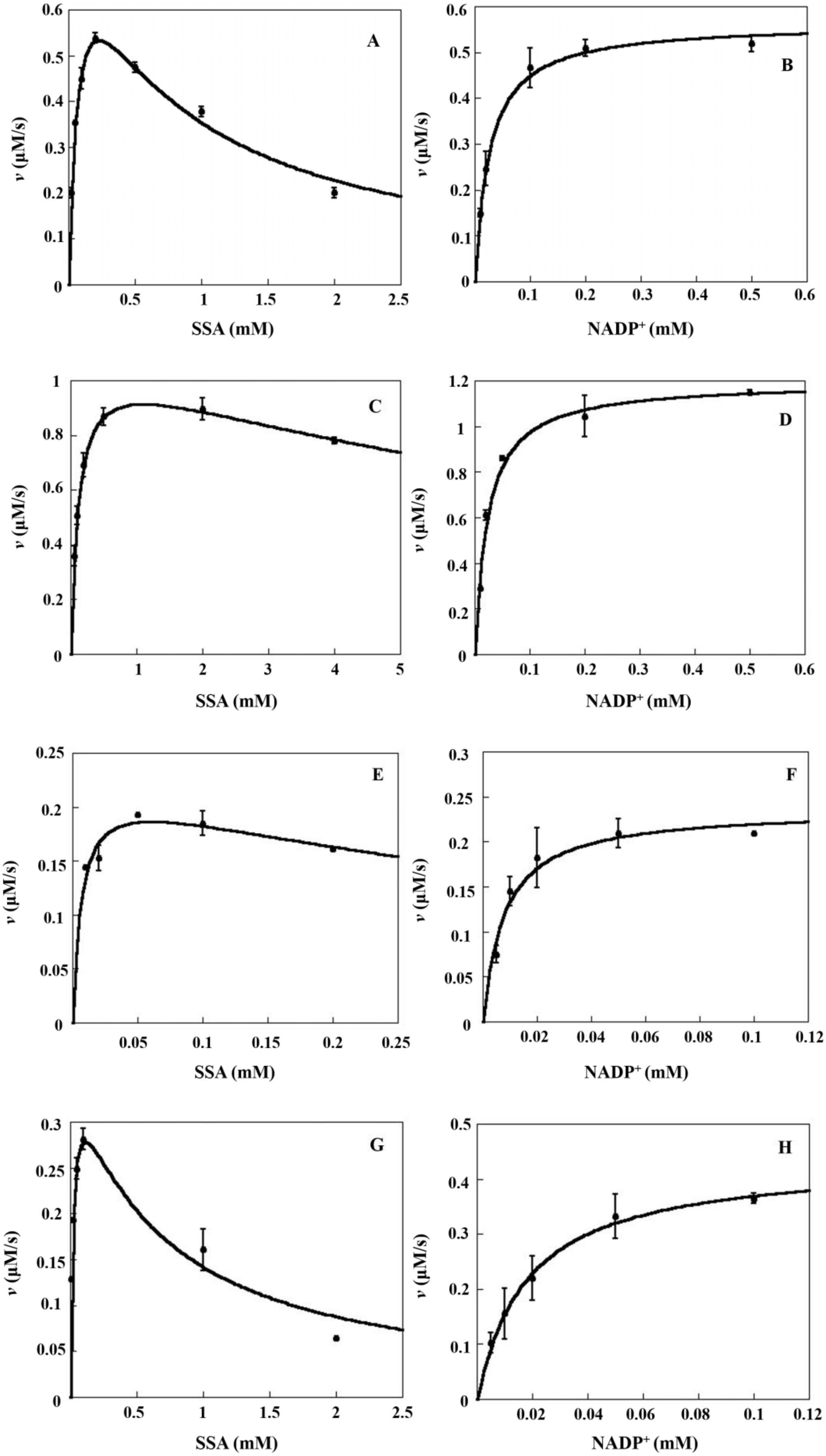
A
v (μM/s)
SSA (mM)
0
0.1
0.2
0.3
0.4
0.5
0.6
0.5
1
1.5
2
2.5
B
v (μM/s)
NADP+ (mM)
0
0.1
0.2
0.3
0.4
0.5
0.6
C
v (μM/s)
SSA (mM)
0
0.2
0.4
0.6
0.8
1
2
3
4
5
D
v (μM/s)
NADP+ (mM)
0
0.2
0.4
0.6
0.8
1
1.2
E
v (μM/s)
SSA (mM)
0
0.05
0.1
0.15
0.2
0.25
F
v (μM/s)
NADP+ (mM)
0
0.05
0.1
0.15
0.2
0.25
0.3
0.02
0.04
0.06
0.08
0.1
0.12
G
v (μM/s)
SSA (mM)
0
0.05
0.1
0.15
0.2
0.25
0.3
0.5
1
1.5
2
2.5
H
v (μM/s)
NADP+ (mM)
0
0.1
0.2
0.3
0.4
0.5
0.02
0.04
0.06
0.08
0.1
0.12

**S6 Fig.** The kinetic analysis of cce4228 mutants.

A: The initial velocities as function of SSA concentrations for S420A with the concentration of $NADP^+$ fixed at 0.5 mM;

B: The initial velocities as function of $NADP^+$ concentrations for S420A with the concentration of SSA fixed at 0.2 mM;

C: The initial velocities as function of SSA concentrations for R139A with the concentration of $NADP^+$ fixed at 0.5 mM;

D: The initial velocities as function of $NADP^+$ concentrations for R139A with the concentration of SSA fixed at 1 mM;

E: The initial velocities as function of SSA concentrations for S207A with the concentration of $NADP^+$ fixed at 0.5 mM;

F: The initial velocities as function of $NADP^+$ concentrations for S207A with the concentration of SSA fixed at 0.05 mM;

G: The initial velocities as function of SSA concentrations for W135A with the concentration of $NADP^+$ fixed at 0.2 mM;

H: The initial velocities as function of $NADP^+$ concentrations for W135A with the concentration of SSA fixed at 0.2 mM.

The above experiments were repeated 3 times.

| | | | | % identity |
|---|---|---|---|---|
| **cce4228** | 254 | RMLNNGQSCIAAKRFILAESIA | 275 | 100 |
| **all3556** | 254 | RMLNNGQSCIAAKRFIVAEAIA | 275 | 73 |
| **a2771** | 254 | RTMNNGQSCIAAKRFILHEAIA | 275 | 65 |
| ***S.typhimurium*** | 260 | RYQNTGQVCAAAKRFIVEEGIA | 281 | 47 |
| **YneI_*E.coli*** | 282 | KFRNAGQVCVSVNRFYIHESVY | 303 | 36 |
| **GabD_*E.coli*** | 281 | KFRNAGQTCVCANRLYVQDGVY | 302 | 34 |
| ***Human*** | 332 | KFRNTGQTCVCSNQFLVQRGIH | 353 | 32 |
| ***A.thaliana*** | 323 | KFRNSGQTCVCANRVLVQDGIY | 344 | 36 |
| ***B.subtilis*** | 258 | KYRNAGQTCVCANRLIVHESIK | 279 | 35 |

**S7 Fig.** The sequence alignment shows that SSADHs have one or two conserved cysteines in their active sites.

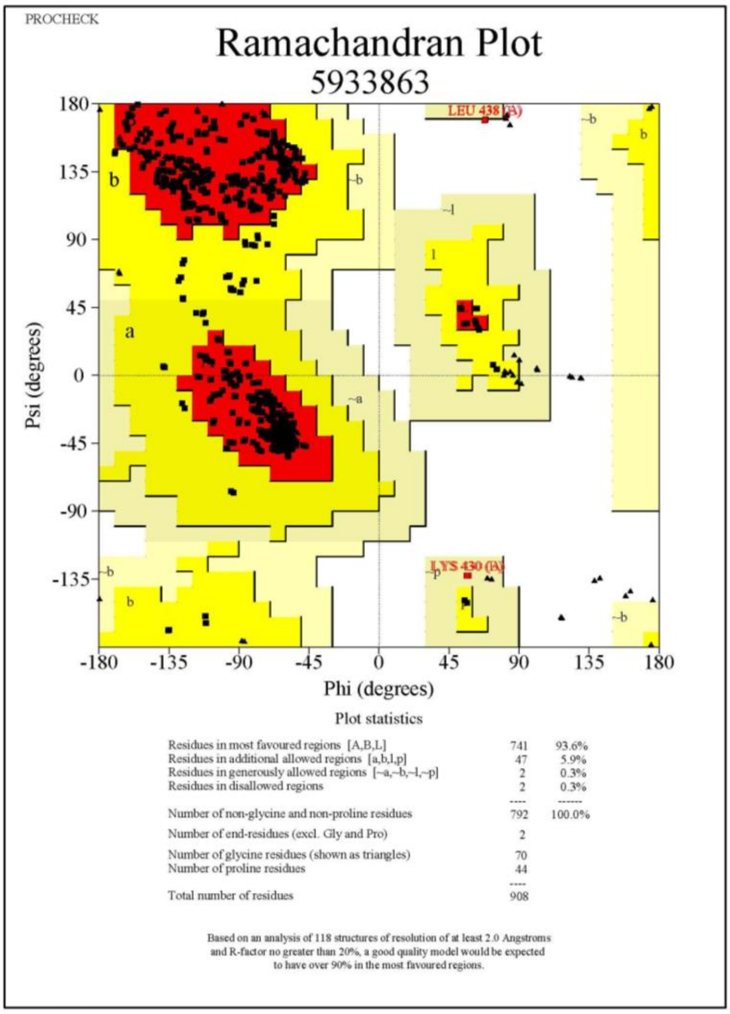


**S8 Fig.** The Ramachandran Plot of the model structure of cce4228 protein. The black rectangle and triangle indicated residues; the most favored regions, additional allowed regions, generously allowed regions and disallowed regions were indicated by red, yellow, light yellow and white, respectively.
